# Clinical Features and Gene Mutation of Neonatal Seizures

**DOI:** 10.3390/jcm15134863

**Published:** 2026-06-23

**Authors:** Liu Guo, Anran Du, Lei An, Guoqian Ding

**Affiliations:** 1 Pediatric Department, Beijing Friendship Hospital, Capital Medical University, Beijing 100050, China; 2 Department of General Surgery, Beijing Friendship Hospital, Capital Medical University, Beijing 100050, China

**Keywords:** neonatal seizures, KCNQ2 gene mutation, subtle seizures, neonatal hypoxic-ischemic encephalopathy, whole-exome sequencing

## Abstract

**Background:** Early-stage seizures caused by an undetermined etiology cannot be treated with effective antiepileptic drugs in a timely manner, leading to poor seizure control and severely affecting long-term prognosis. Therefore, early clarification of the cause and targeted timely treatment are crucial. **Objective:** To characterize the clinical features and genetic mutations in neonatal seizures, particularly those of unknown cause. **Methods:** Clinical data from 56 neonates with seizures were retrospectively analyzed. Family-based whole-exome sequencing was performed in six cases of undetermined etiology. The KCNQ2 variant was classified according to the ACMG guidelines. Basic statistical comparisons were performed using the chi-square test. **Results:** Hypoxic-ischemic encephalopathy (HIE) was the most common cause (23.21%, 13/56), with most seizures occurring within the first three days of life. Subtle seizures were the predominant type (46.42%, 26/56). Abnormal amplitude-integrated EEG findings were observed in 58.82% (30/51). The HIE group had a significantly higher proportion of seizures occurring within the first three days of life compared to the non-HIE group (*p* = 0.01). A novel heterozygous KCNQ2 mutation (c.766G>T, p.Gly256Trp) was identified and classified as likely pathogenic according to the ACMG guidelines. **Conclusions:** HIE remains a leading cause of early-onset neonatal seizures. A novel likely pathogenic KCNQ2 mutation expands the genetic spectrum of neonatal seizures, highlighting the value of genetic testing.

## 1. Introduction

Neonatal seizures are the most common clinical manifestation of central nervous system dysfunction in the first month of life, affecting 1–5 per 1000 live births [1]. The immature neonatal brain has heightened excitability in subcortical centers and poor inhibitory function, making it uniquely vulnerable to abnormal discharges [2]. Unlike in older children and adults, neonatal seizures often present with subtle or subclinical manifestations, complicating early diagnosis [3].

Etiologies of neonatal seizures are highly heterogeneous and include hypoxic-ischemic encephalopathy (HIE), intracranial infections, intracranial hemorrhage, genetic metabolic disorders, cerebral maldevelopment, and benign familial neonatal seizures [4]. HIE remains the predominant cause worldwide, accounting for 30–50% of cases in most cohorts [5]. However, the relative proportion of genetic and metabolic causes has increased in recent years due to advances in tandem mass spectrometry and next-generation sequencing technologies [6].

Despite thorough clinical evaluation, some cases remain etiologically undetermined, delaying targeted treatment and worsening long-term prognosis [7]. Genetic testing has increasingly clarified such cases. Shellhaas et al. reported that 43% of infants with neonatal epilepsy have identifiable genetic etiologies, with the rate rising to 83% in epileptic encephalopathies [8]. Among genetic causes, KCNQ2 mutations are among the most frequently identified, encoding a voltage-gated potassium channel subunit critical for regulating neuronal excitability [9].

KCNQ2-related disorders span a clinical spectrum from self-limited (benign) familial neonatal epilepsy (BFNE) to severe developmental and epileptic encephalopathy (DEE) [10]. The distinction between these phenotypes has profound implications for prognosis and treatment. BFNE typically carries a favorable outcome, with seizures resolving by early infancy, whereas KCNQ2-DEE often results in severe developmental delay and intellectual disability [11]. Therefore, early genetic diagnosis is crucial for guiding management and counseling families.

This study retrospectively analyzes clinical data from 56 neonates with seizures, describes the etiological distribution and clinical features, and investigates genetic mutations in unexplained cases. Particular attention is given to methodological transparency, cautious interpretation of genetic findings, and discussion of study limitations.

## 2. Materials and Methods

### 2.1. Study Design and Participants

This retrospective study included 56 neonates with seizures who were hospitalized in the Department of Neonatology, Beijing Friendship Hospital, between July 2015 and May 2024.

Inclusion criteria: (1) Diagnosis of neonatal seizures based on clinical manifestations and/or electroencephalogram (EEG) findings; (2) hospitalization and treatment in our department; (3) complete clinical and follow-up data.

Exclusion criteria: (1) Severe congenital heart disease or other systemic diseases affecting prognosis; (2) death immediately after severe perinatal asphyxia; (3) incomplete medical records.

Among the 56 included patients, there were 32 males and 24 females, including 41 full-term and 15 preterm infants. This study was approved by the Ethics Committee of Beijing Friendship Hospital, Capital Medical University (BFHHZS20220301). Written informed consent was obtained from the guardians of all participants, including consent for genetic testing and publication of anonymized data.

Diagnostic criteria for the etiologies were defined as follows:

- Hypoxic-ischemic encephalopathy (HIE): Perinatal asphyxia (Apgar score ≤ 5 at 5 min, cord blood pH < 7.0 or base deficit ≥ 16 mmol/L) plus clinical encephalopathy, staged per the Sarnat criteria.
- Intracranial infection: Positive cerebrospinal fluid culture or polymerase chain reaction (PCR), or pleocytosis with elevated protein and hypoglycorrhachia.
- Intracranial hemorrhage: Confirmed by cranial ultrasound or magnetic resonance imaging (MRI).
- Acute metabolic disorders: Hypoglycemia (blood glucose < 2.6 mmol/L) or hyperbilirubinemia (total bilirubin exceeding the exchange transfusion threshold).
- Congenital genetic metabolic diseases: Confirmed by tandem mass spectrometry, enzyme assays, or genetic testing for specific disorders (e.g., pyridoxine dependency, hyperammonemia).

### 2.2. Seizure Classification and EEG

Seizure types were classified according to the International League Against Epilepsy (ILAE) 2021 neonatal seizure classification [12] based on simultaneous video-EEG monitoring (NicoletOne, 30–120 min, Natus Neurology Incorporated, Middleton, WI, USA). Amplitude-integrated EEG (aEEG) was used for bedside continuous monitoring (Olympic Brainz Monitor, 24–72 h, Natus Neurology Incorporated, Middleton, WI, USA) but was not used as the sole basis for seizure type classification, given its known limitations in detecting focal or subtle seizures [13].

### 2.3. Treatment Protocol and Response Definition

Phenobarbital was the first-line treatment, administered intravenously at an initial loading dose of 20 mg/kg, followed by a maintenance dose of 5 mg/kg/day in two divided doses after 12 h. Intravenous midazolam was used for non-responders (loading dose 0.1–0.3 mg/kg, maintenance infusion starting at 1.0 µg/kg/min, titrated every 15 min up to a maximum of 8 µg/kg/min). Alternatively, oral levetiracetam was given (initial dose 30 mg/kg, repeated after 8 h if needed, then maintenance at 15 mg/kg twice daily).

Treatment ‘effective’ was defined as cessation of clinical seizures within 72 h of initiating phenobarbital monotherapy, confirmed by EEG (no electrographic seizures for ≥24 h). Only 22 patients receiving phenobarbital monotherapy for ≥72 h with complete clinical and EEG data were included in the treatment-response analysis. The remaining 34 patients were excluded due to immediate seizure control after treating acute metabolic causes (*n* = 12), use of other first-line agents (*n* = 8), combination therapy initiated before 72 h (*n* = 7), self-discharge or loss to follow-up (*n* = 5), or incomplete EEG data (*n* = 2).

### 2.4. Genetic Testing and Whole-Exome Sequencing

Of the 16 cases with undetermined etiology after an initial routine evaluation, six underwent family-based whole-exome sequencing (WES). The selection criteria for WES is as follows: (1) parental consent for trio analysis; (2) absence of acquired causes after a comprehensive metabolic, infectious, and neuroimaging workup; (3) clinical suspicion of genetic etiology (e.g., family history of neonatal seizures or epilepsy, dysmorphic features, treatment resistance, normal metabolic and infectious workup).

Peripheral blood samples (3 mL each) were collected from the six neonates and their parents. WES was performed on the Illumina NovaSeq 6000 platform (Illumina, Inc., San Diego, CA, USA), achieving an average sequencing depth of >100× and >99% of target regions covered at least 20×. The whole-exome V1 probe kit (Integrated DNA Technologies, Coralville, IA, USA)was used for target capture. Raw sequencing data were aligned to the UCSC hg19 human reference genome using BWA-MEM, and variants were called using GATK HaplotypeCaller (Illumina, San Diego, CA, USA).

Limitations of WES in this study: The WES protocol used does not reliably detect copy-number variants (CNVs), mitochondrial DNA variants, deep intronic variants, or structural variants (e.g., balanced translocations, inversions). These limitations are acknowledged as potential explanations for negative genetic findings in the remaining five cases.

### 2.5. Variant Filtering and Interpretation

Variants were annotated using ANNOVAR (20211019) and filtered against population databases (gnomAD v2.1, 1000 Genomes Project phase 3, dbSNP v151). Non-synonymous, splice-site (±10 bp), and indel variants with a minor allele frequency <1% were prioritized. Pathogenicity was assessed according to the American College of Medical Genetics and Genomics (ACMG) 2015 guidelines [14]. The following evidence codes were used for the KCNQ2 variant: PM2 (absent from controls in gnomAD), PS2 (confirmed de novo with both parents wild type), PP3 (multiple in silico tools predict damaging), PM1 (located in a mutational hotspot, the pore loop), or PP2 (missense variant in a gene with a low rate of benign missense variation). Pathogenic or likely pathogenic variants were validated by Sanger sequencing in the family context.

Functional validation (e.g., patch-clamp electrophysiology to assess potassium channel function) was not performed, which represents a major limitation in definitively establishing pathogenicity.

### 2.6. Bioinformatics Analysis

Functional impact of point mutations was predicted using SIFT (v6.2.0), MutationTaster (v2021), and PhD-SNPg (v2019). Conservation analysis across species was performed using UniProt alignment. PyMOL (v2.5) was used to analyze spatial structural differences in the KCNQ2 protein before and after mutation using the published cryo-EM structure of Kv7.2 (PDB: 7CR0) as a template.

### 2.7. Statistical Analysis

Statistical analysis was performed using SPSS version 26.0 (IBM Corp., Armonk, NY, USA). Descriptive statistics (frequencies and percentages) were used to summarize the clinical characteristics. The chi-square test (or Fisher‘s exact test where expected cell counts were <5) was used for comparative analyses between groups (e.g., HIE vs. non-HIE) for categorical variables. A two-tailed *p*-value < 0.05 was considered statistically significant.

## 3. Results

### 3.1. General and Clinical Characteristics

Among 56 patients (32 male, 24 female; 41 full-term, 15 preterm), HIE was the most commonly identified cause (23.21%, 13/56), followed by congenital genetic metabolic diseases (14.28%, 8/56) and intracranial infection (12.50%, 7/56). Notably, 28.57% (16/56) of the cases remained undetermined after an initial routine clinical evaluation (Table 1).

Seizure onset occurred within the first three days of birth in 62.50% (35/56), between 4–7 days in 25.00% (14/56), and after one week in 12.50% (7/56). Subtle seizures were the most common type (46.42%, 26/56), followed by myoclonic (23.21%, 13/56), clonic (19.64%, 11/56), tonic (7.14%, 4/56), and mixed seizure types (3.57%, 2/56).

EEG abnormalities were observed in 58.82% (30/51; five patients did not undergo EEG). Of these, 20 patients showed epileptiform discharges, 6 had abnormal background activity, and 4 exhibited periodic abnormalities (Table 2).

### 3.2. Comparative Analysis: HIE vs. Non-HIE

To further explore clinical associations, we compared the HIE group (*n* = 13) with the non-HIE group (*n* = 43). As shown in Table 3, the HIE group had a significantly higher proportion of seizure onset within the first three days of life (92.31% vs. 53.49%, *p* = 0.01). EEG abnormalities were also more frequent in the HIE group (84.62% vs. 51.16%, *p* = 0.03).

### 3.3. Treatment Outcomes

Among the 22 patients receiving phenobarbital monotherapy with complete data, 15 (68.18%) met the predefined criteria for treatment ‘effective’ (clinical seizure cessation within 72 h, confirmed by EEG). The remaining seven patients required additional anticonvulsants: three received levetiracetam plus phenobarbital, two received intravenous midazolam, and two were lost to follow-up after self-discharge.

### 3.4. Whole-Exome Sequencing Analysis and Protein Structure Modeling

**Figure 1:**
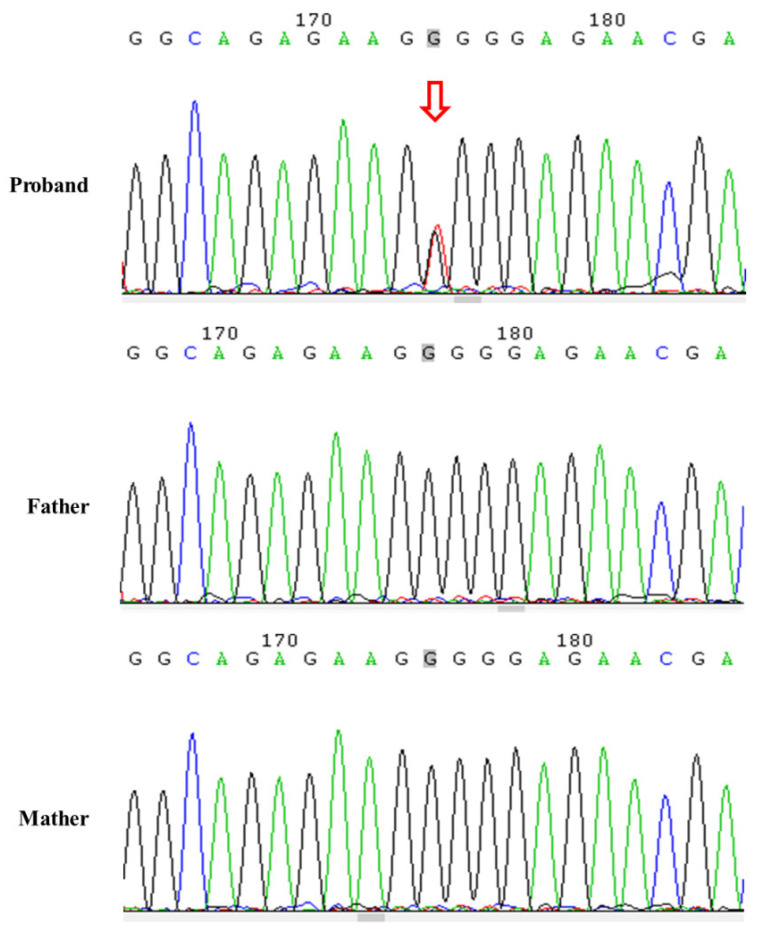
Whole-exome sequencing results showing a mutation in the *KCNQ2* gene, c.766G>T, p.Gly256Trp (NM_172107.3), with both parents being wild type. Sanger sequencing confirmed the presence of this mutation (red arrow).

Of the six undetermined cases undergoing whole-exome sequencing (WES), one patient harbored a heterozygous missense variant in the *KCNQ2* gene: *c.766G>T*, *p.Gly256Trp* (NM_172107.3). Sanger sequencing confirmed the presence of this mutation, while both parents showed a normal *KCNQ2* gene (Figure 1). The dbSNP database, the 1000 Genomes database, and the gnomAD database did not include this variant, suggesting that it is a novel mutation.

**Figure 2:**
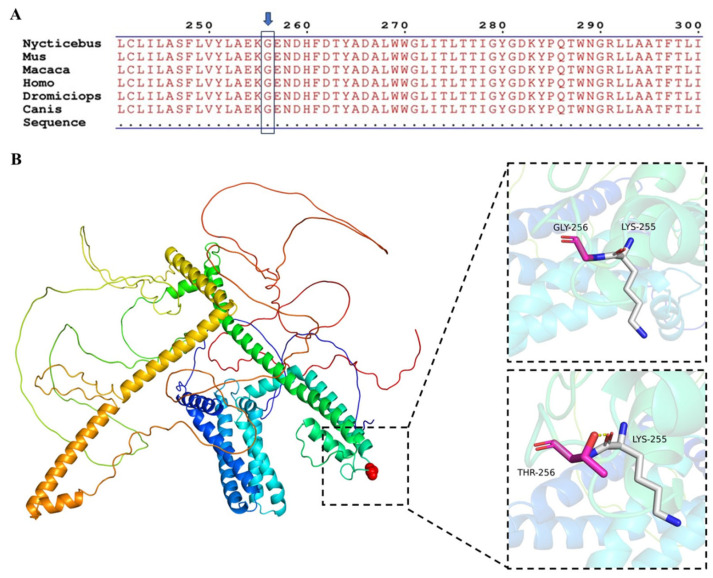
*KCNQ2* protein *Gly256* mutation conservation and structural prediction analysis. (**A**) *KCNQ2* protein *Gly256* shows high conservation across multiple species (blue arrow); (**B**) Protein structure prediction showing that the Gly256Trp mutation may lead to the formation of a hydrogen bond with Lys255.

In silico predictions using SIFT (score 0.043, damaging), MutationTaster (score 0.999, disease causing), and PhD-SNPg (score 0.963, pathogenic) all suggested deleterious effects. Homology alignment showed that Gly256 of the KCNQ2 protein is highly conserved across species from zebrafish to humans, indicating functional importance (Table 4). PyMOL-based structural modeling using the Kv7.2 cryo-EM structure (PDB: 7CR0) suggested that the substitution of non-polar glycine with polar tryptophan at position 256 may lead to local conformational changes in the pore loop (P-loop) and potential hydrogen bond formation with Lys255 (Figure 2). However, these in silico predictions require electrophysiological confirmation.

### 3.5. Clinical and Genetic Findings of the Six Patients Who Underwent WES

The clinical and genetic summary of six patients undergoing WES is shown in Table 5. The patient (P1 in Table 5) was a full-term male born to non-consanguineous parents with no family history of seizures or epilepsy. He presented on day 2 of life with recurrent episodes of cyanosis, upper limb flexion posturing, and lower limb rigidity, each lasting 20–30 s and occurring 10–12 times daily. Interictal video-EEG showed a burst-suppression pattern with multifocal epileptiform discharges. Ictal EEG revealed electrodecremental events followed by rhythmic alpha-beta activity.

The patient’s cranial MRI (3T) was normal. Extensive metabolic testing (plasma amino acids, urine organic acids, blood acylcarnitine profile, CSF neurotransmitters) was unremarkable. The patient did not respond to phenobarbital (20 mg/kg load, 5 mg/kg/day maintenance) after 72 h. Levetiracetam was added (initial 30 mg/kg, maintenance 15 mg/kg twice daily), achieving seizure freedom within 48 h. At 2 years of age, the patient has remained seizure-free, is off all antiseizure medications, and has demonstrated normal neurodevelopmental outcomes (Gesell Developmental Scale score: 92, age-appropriate for all domains). This phenotype is most consistent with self-limited (benign) familial neonatal epilepsy rather than KCNQ2-related developmental and epileptic encephalopathy.

## 4. Discussion

### 4.1. Etiological Distribution and Clinical Features

In this single-center retrospective cohort of 56 neonates with seizures, HIE remained the predominant identified cause, accounting for 23.21% of cases. This finding is consistent with previous large-scale studies reporting HIE as the leading cause of neonatal seizures worldwide, with proportions ranging from 30% to 50% in resource-rich settings [14]. The strong association we observed between HIE and seizure onset within the first three days of life (*p* = 0.01) aligns with the well-established pathophysiology of perinatal asphyxia, in which brain injury evolves over the first 24–72 h after the hypoxic-ischemic insult [12].

Subtle seizures were the most common seizure type (46.42%), which is in line with the literature [12]. This predominance reflects the unique neurodevelopmental state of the neonatal brain: incomplete myelination, immature dendritic arborization, and relative hyperexcitability of subcortical structures limit the synchronization and propagation of cortical discharges, making tonic-clonic seizures rare [2]. The high proportion of subtle seizures underscores the clinical challenge of seizure recognition in neonates and the critical role of EEG monitoring.

### 4.2. Genetic Findings: A Candidate KCNQ2 Variant

The identification of a de novo *KCNQ2* missense variant (c.766G>T, p.Gly256Trp) in one patient with unexplained neonatal seizures represents the most novel finding of this study. However, several important caveats must be emphasized. First, the variant is classified as a Variant of Uncertain Significance (VUS) under ACMG 2015 guidelines [14], not as pathogenic. While multiple lines of in silico evidence and the de novo status support potential pathogenicity, definitive classification would require functional validation [15].

Second, the location of this variant in the pore loop (P-loop) between transmembrane segments S5 and S6 is intriguing, as this region forms the selectivity filter of the voltage-gated potassium channel [16]. Previously reported pathogenic *KCNQ2* mutations in the pore loop (e.g., G256S, G256R, W236R) have been associated with a range of phenotypes from self-limited neonatal epilepsy to severe encephalopathy [17,18]. The Gly256 residue is invariant across species from zebrafish to humans, suggesting strong evolutionary conservation of function. However, the specific amino acid substitution (Gly→Trp) observed in our patient has not been previously reported, and its functional consequences remain unknown.

Third, the clinical phenotype of our patient—early-onset seizures, burst-suppression EEG, favorable response to levetiracetam, and normal neurodevelopment at 2 years—is most consistent with self-limited (benign) familial neonatal epilepsy (BFNE), a condition typically associated with *KCNQ2* loss-of-function mutations [19]. This contrasts with *KCNQ2*-related DEE, which presents with more severe, treatment-resistant seizures and poor developmental outcomes. The favorable outcome in our patient, despite the burst-suppression pattern (often considered a poor prognostic sign), highlights the phenotypic heterogeneity of *KCNQ2*-related disorders and the importance of genetic diagnosis for prognostication [20].

### 4.3. Oxidative Stress and Neuroinflammation in Neonatal Seizures

Beyond genetic mechanisms, neonatal seizures themselves can trigger secondary injury cascades. Experimental studies have demonstrated that prolonged or recurrent neonatal seizures activate NOX2/NADPH oxidase, leading to reactive oxygen species (ROS) overproduction and subsequent oxidative damage to lipids, proteins, and DNA [21]. This oxidative stress, in turn, impairs the nuclear factor erythroid 2-related factor 2 (Nrf2) antioxidant response pathway, reducing the expression of cytoprotective enzymes such as glutathione S-transferase and NAD(P)H quinone oxidoreductase 1 [22].

Moreover, seizure-induced ROS can activate pro-inflammatory transcription factors, including NF-κB and AP-1, promoting the release of cytokines such as IL-1β, IL-6, and TNF-α [23]. This neuroinflammatory response may contribute to acute brain injury and, over the long term, facilitate epileptogenesis—the process by which a normal brain becomes epileptic [24]. Although we did not directly measure these biomarkers in our cohort, understanding these pathways provides a mechanistic framework that contextualizes our clinical observations and suggests potential neuroprotective strategies (e.g., Nrf2 activators, antioxidants) that warrant investigation in future studies.

### 4.4. Network-Level Biomarkers in Genetic Epilepsies

Recent advances in EEG signal analysis have identified high-frequency oscillations (HFOs), particularly fast ripples in the 250–500 Hz range, as potential biomarkers of epileptogenicity and epileptogenesis [25]. In genetic epilepsies, including those caused by *KCNQ2* mutations, *HFOs* may be detectable on a scalp or intracranial EEG before the appearance of conventional epileptiform discharges [17]. Fast ripples are thought to reflect the synchronous firing of pathologically interconnected neuron clusters—a hallmark of an epileptic network [26].

While we did not perform *HFO* analysis in this study, this approach represents a promising direction for future research. Integrating *HFO* detection with genetic testing could improve genotype–phenotype correlations, identify patients at risk for DEE before developmental regression occurs, and provide objective biomarkers for treatment response [27]. The burst-suppression pattern observed in our *KCNQ2*-positive patient is known to be associated with *HFOs*, and retrospective analysis of stored EEG data for HFOs is planned as part of ongoing work.

### 4.5. KCNQ2-Related Epilepsy: Genotype–Phenotype Correlations

The *KCNQ2* gene encodes the *Kv7.2* subunit, which co-assembles with *Kv7.3* (encoded by *KCNQ3*) to form the M-current—a slowly activating, non-inactivating potassium current that regulates neuronal excitability by stabilizing the resting membrane potential [28]. Loss-of-function mutations in *KCNQ2* reduce M-current amplitude, leading to hyperexcitability and seizure susceptibility. However, the severity of functional impairment correlates roughly with clinical severity: BFNE-associated mutations typically cause partial loss of function (20–60% residual current), whereas DEE-associated mutations often result in near-complete loss-of-function or dominant-negative effects [29].

Our patient’s mutation (*p.Gly256Trp*) is located in the pore loop, a region critical for ion selectivity and conductance. Previous studies of pore-loop mutations in *KCNQ2* have shown variable electrophysiological effects. For example, *G256S* resulted in ~30% residual current and was associated with *BFNE* [17], whereas *G256R* caused near-complete loss of function and was associated with *DEE* [18]. Without patch-clamp data, we cannot determine whether *p.Gly256Trp* behaves similarly to *G256S* or *G256R*. The favorable clinical outcome in our patient suggests, but does not prove, a milder functional defect. This uncertainty underscores the essential need for functional validation in future studies.

### 4.6. Comparison with Published Cohorts

The proportion of undetermined cases in our cohort (28.57%) is comparable to that reported in other single-center studies before the widespread adoption of genetic testing [7]. However, this figure is higher than in recent studies incorporating routine genetic testing [8], suggesting that some of our undetermined cases might have identifiable genetic etiologies. Among the five WES-negative patients, potential explanations include (1) pathogenic variants in genes not covered effectively by our WES (e.g., CNVs, mitochondrial DNA, deep intronic variants); (2) variants in genes not yet associated with neonatal seizures; or (3) non-genetic etiologies not identified by routine testing.

The relatively high proportion of genetic metabolic diseases (14.28%) in our cohort may reflect referral bias to a tertiary center or local diagnostic practice, rather than true population epidemiology. Similar overrepresentation of metabolic cases has been noted in other single-center studies from China [3].

### 4.7. Clinical Implications and Practice Recommendations

Based on our findings and current literature, we suggest the following practice considerations for neonates with unexplained seizures:

1. Early genetic testing: WES or targeted gene panels should be considered early in the diagnostic workup of neonates with unexplained seizures, particularly for those with treatment resistance, a normal metabolic and infectious workup, or suggestive family history.
2. Cautious variant interpretation: Novel KCNQ2 variants should be classified using ACMG guidelines, and VUS designations should be clearly communicated to families. Functional validation should be pursued when possible.
3. Seizure recognition: Given the predominance of subtle seizures, continuous video-EEG monitoring is preferred over aEEG alone for diagnosis and treatment monitoring.
4. Prognostic counseling: In KCNQ2-related disorders, genotype alone does not perfectly predict outcome. Even patients with early burst-suppression patterns may have favorable outcomes, as illustrated by our patient.

### 4.8. Limitations

This study has substantial limitations that must be acknowledged.

Study design limitations: The retrospective, single-center design with a small sample size (*n* = 56) limits generalizability. The etiological distribution, including the relatively high proportion of undetermined (28.57%) and metabolic cases (14.28%), may reflect referral patterns to our tertiary center rather than broader neonatal seizure epidemiology. The lack of a validation cohort precludes external validation of our findings.

Selection bias in genetic testing: Only 6 of the 16 undetermined cases underwent WES, based on clinical suspicion and consent. This selective approach may overestimate the diagnostic yield of genetic testing and does not represent a true incidence rate. The remaining 10 undetermined cases without genetic testing represent a missed opportunity for etiological diagnosis.

Genetic limitations: The *KCNQ2* variant is classified as *VUS*, not pathogenic. Definitive pathogenicity would require functional validation (e.g., patch-clamp electrophysiology in heterologous expression systems), which was not performed. Additionally, the WES protocol used does not reliably detect CNVs, mitochondrial DNA variants, deep intronic variants, or structural variants, any of which could explain the negative results in the remaining five patients.

Outcome limitations: Systematic developmental follow-up data are available for only a subset of patients (including the KCNQ2-positive patient). We lack standardized neurodevelopmental assessments for the full cohort at consistent time points, precluding robust conclusions about long-term prognosis.

EEG limitations: Although video-EEG was used for classification, aEEG was used for bedside monitoring. aEEG has well-documented lower sensitivity for focal, subtle, or brief seizures compared to continuous video-EEG. Some seizures may therefore have been missed, potentially affecting treatment-response assessments.

Absence of mechanistic biomarkers: We did not measure oxidative stress markers (e.g., 8-OHdG, malondialdehyde), inflammatory cytokines, or HFOs. Incorporating these biomarkers into future studies could strengthen genotype–phenotype correlations and identify novel therapeutic targets.

## 5. Conclusions

In this single-center retrospective cohort of 56 neonates with seizures, HIE remains the most common identifiable cause, with seizure onset typically occurring within the first three days of life. Subtle seizures are the predominant clinical presentation, highlighting the essential role of EEG monitoring for accurate diagnosis.

A candidate de novo *KCNQ2* missense variant (c.766G>T, p.Gly256Trp) was identified in one unexplained case but remains a Variant of Uncertain Significance (VUS) in the absence of functional validation. The patient’s favorable clinical outcome (seizure freedom, normal development at 2 years) is most consistent with self-limited neonatal epilepsy rather than KCNQ2-related DEE. The claim that this variant “may lead to neonatal seizures” is preliminary and requires confirmation through electrophysiological studies.

Future studies should incorporate the following: (1) multicenter prospective designs with standardized protocols; (2) routine genetic testing (WES or genome sequencing) for all undetermined cases; (3) functional validation of novel variants (patch-clamp, neuronal models); (4) systematic long-term neurodevelopmental follow-up; and (5) exploration of mechanistic biomarkers (oxidative stress, inflammation, HFOs). These approaches will advance precision medicine for neonates with seizures of undetermined etiology.

## Figures and Tables

**Table 1:** Etiology of neonatal seizures (N = 56).

Etiology	Number	Proportion (%)
Undetermined	16	28.57
Hypoxic-ischemic encephalopathy (HIE)	13	23.21
Congenital genetic metabolic diseases	8	14.28
Intracranial infection	7	12.50
Intracranial hemorrhage	6	10.71
Acute metabolic disorders (hypoglycemia, hyperbilirubinemia)	4	7.14
Cerebral maldevelopment	1	1.79
Cerebral infarction	1	1.79

**Table 2:** Clinical features and EEG findings.

Feature	Number	Proportion (%)
Time of first seizure onset		
<3 days	35	62.50
4–7 days	14	25.00
>1 week	7	12.50
Seizure type		
Subtle	26	46.42
Myoclonic	13	23.21
Clonic	11	19.64
Tonic	4	7.14
Mixed (≥2 types)	2	3.57
EEG findings (*n* = 51)		
Abnormal background	6	11.76
Periodic abnormalities	4	7.84
Epileptiform discharges	20	39.22
Normal	21	41.18

**Table 3:** Comparative analysis between HIE and non-HIE groups.

Feature	HIE Group (n = 13)	Non-HIE Group (n = 43)	*p*-Value
Seizure onset < 3 days	12 (92.31%)	23 (53.49%)	0.01
Abnormal EEG findings	11 (84.62%)	22 (51.16%)	0.03

**Table 4:** KCNQ2 gene mutation site pathogenicity prediction.

Nucleotide Change	Mutation Type	Prediction Tool	Score	Prediction
c.766G>T	Missense mutation	SIFT	0.043	Damaging
		MutationTaster	0.999	Disease causing
		PhD-SNPg	0.963	Pathogenic

SIFT: Sorting Intolerant From Tolerant.

**Table 5:** Clinical and genetic summary of six patients undergoing whole-exome sequencing. Abbreviations: LEV, levetiracetam; PB, phenobarbital; TPM, topiramate; MDZ, midazolam; VUS, variant of uncertain significance; N/A, not applicable.

Patient ID	Onset Day	Seizure Type	EEG Pattern	Brain MRI	Metabolic Screen	Treatment	Gene (Variant)	ACMG Classification	Outcome at 2 Years
P1	D2	Tonic-clonic	Burst-suppression	Normal	Normal	LEV → seizure-free	KCNQ2 c.766G>T p.Gly256Trp	VUS (supporting pathogenic)	Normal development (Gesell 92)
P2	D5	Subtle	Multifocal spikes	Normal	Normal	PB → seizure-free	Negative	N/A	Normal
P3	D1	Myoclonic	Hypsarrhythmia	Normal	Normal	PB + TPM → partial	Negative	N/A	Mild delay (Gesell 78)
P4	D3	Clonic	Focal spikes	Normal	Normal	PB + LEV → seizure-free	Negative	N/A	Normal
P5	D7	Subtle	Normal	Normal	Normal	PB → seizure-free	Negative	N/A	Normal
P6	D2	Tonic	Burst-suppression	Normal	Normal	PB + MDZ → seizure-free	Negative	N/A	Lost to follow-up

## Data Availability

The data presented in this study are available on reasonable request from the corresponding author due to privacy and ethical restrictions.

## References

[1] Ziobro J., Shellhaas R.A. (2020). Neonatal Seizures: Diagnosis, Etiologies, and Management. Semin. Neurol..

[2] Samanci S., Celik M., Akdeniz O., Deger I., Ozgun N., Kanar B., Tuzun H. (2023). The role of metabolic diseases in neonatal convulsions. Eur. Rev. Med. Pharmacol. Sci..

[3] Spagnoli C., Falsaperla R., Deolmi M., Corsello G., Pisani F. (2018). Symptomatic seizures in preterm newborns: A review on clinical features and prognosis. Ital. J. Pediatr..

[4] Pisani F., Spagnoli C., Falsaperla R., Nagarajan L., Ramantani G. (2021). Seizures in the neonate: A review of etiologies and outcomes. Seizure.

[5] Judy R.L., Reynolds J.L., Jnah A.J. (2024). Identifying Metabolic Diseases That Precipitate Neonatal Seizures. Neonatal Netw..

[6] Akiyama L.F., Saneto R.P. (2023). Early-Life Epilepsies. Pediatr. Ann..

[7] Sandoval K.A., DiGiovine M.P., Massey S.L. (2024). Neonatal Seizures. Pediatr. Rev..

[8] Wong S.-H., Liou Y.-M., Yang J.-J., Lee I.-C. (2024). KCNQ2 mutations cause unique neonatal behavior arrests without motor seizures: Functional characterization. Epilepsy Behav..

[9] Huang Z., Liu B., Xiao T., Wang Y., Lu Y., Hu L., Cheng G., Li Z., Wang L., Zhang R. (2024). Neurodevelopmental Outcomes Prediction in Newborns with Seizures Caused by KCNQ2 Gene Defects. Neonatology.

[10] Bonardi C.M., Heyne H.O., Fiannacca M., Fitzgerald M.P., Gardella E., Gunning B., Olofsson K., Lesca G., Verbeek N., Stamberger H. (2021). KCNT1-related epilepsies and epileptic encephalopathies: Phenotypic and mutational spectrum. Brain.

[11] Zimmern V., Korff C. (2022). Updates on the diagnostic evaluation, genotype-phenotype correlation, and treatments of genetic epilepsies. Curr. Opin. Pediatr..

[12] Pressler R.M., Cilio M.R., Mizrahi E.M., Moshé S.L., Nunes M.L., Plouin P., Vanhatalo S., Yozawitz E., de Vries L.S., Vinayan K.P. (2021). The ILAE classification of seizures in the neonate. Epilepsia.

[13] Shellhaas R.A. (2016). Amplitude-integrated electroencephalography for neonatal seizure detection: Limitations and utility. J. Clin. Neurophysiol..

[14] Richards S., Aziz N., Bale S., Bick D., Das S., Gastier-Foster J., Grody W.W., Hegde M., Lyon E., Spector E. (2015). Standards and guidelines for the interpretation of sequence variants: A joint consensus recommendation of the American College of Medical Genetics and Genomics and the Association for Molecular Pathology. Genet. Med..

[15] Brnich S.E., Abou Tayoun A.N., Couch F.J., Cutting G.R., Greenblatt M.S., Heinen C.D., Kanavy D.M., Luo X., McNulty S.M., Starita L.M. (2019). Recommendations for application of the functional evidence PS3/BS3 criterion using the ACMG/AMP sequence variant interpretation framework. Genome Med..

[16] Jentsch T.J. (2000). Neuronal KCNQ potassium channels: Physiology and role in disease. Nat. Rev. Neurosci..

[17] Yang G.-M., Tian F.-Y., Shen Y.-W., Yang C.-Y., Yuan H., Li P., Gao Z.-B. (2023). Functional characterization and in vitro pharmacological rescue of KCNQ2 pore mutations associated with epileptic encephalopathy. Acta Pharmacol. Sin..

[18] Zhang J., Zhao J., Zhang X., Wang Y., Li W., Liu X. (2021). KCNQ2 mutations in neonatal epilepsy: Genotype-phenotype correlation and literature review. Front. Neurol..

[19] Sands T.T., Balestri M., Bellini G., Mulkey S.B., Danhaive O., Bakken E.H., Taglialatela M., Soldovieri M.V. (2020). Rapid and sustained response to low-dose levetiracetam in a neonate with KCNQ2-related self-limited epilepsy. Epilepsy Behav. Rep..

[20] Pisani F., Spagnoli C., Pavlidis E., Cantalupo G. (2020). The role of EEG in the diagnosis and prognosis of KCNQ2-related epileptic encephalopathy. Seizure.

[21] Patel M. (2016). Targeting oxidative stress in central nervous system disorders. Trends Pharmacol. Sci..

[22] Johnson J.A., Johnson D.A., Kraft A.D., Calkins M.J., Jakel R.J., Vargas M.R., Zhang J. (2009). The Nrf2-ARE pathway: A potential therapeutic target for neurodegenerative diseases. Antioxid. Redox Signal..

[23] Vezzani A., Balosso S., Ravizza T. (2008). The role of cytokines in the pathophysiology of epilepsy. Brain Behav. Immun..

[24] Pitkänen A., Lukasiuk K., Bhatt K.A., Williams P.A., Pool A.H., Switzer R.C., Sharp F.R. (2021). Neuroinflammation and epileptogenesis: A review of recent experimental studies. Epilepsia.

[25] Sheybani L., Qiu Y., Singh P.K., Vivekananda U., Burgess N., Diehl B., McEvoy A., Miserocchi A., Bisby J.A., Shekh-Ahmad T. (2026). Fast-ripples are emergent properties of neuronal networks. bioRxiv.

[26] Köhling R., Staley K. (2011). Network mechanisms for fast ripple activity in epileptic tissue. Epilepsy Res..

[27] Shear M.A., Robinson P.N., Sparks T.N. (2025). Fetal imaging, phenotyping, and genomic testing in modern prenatal diagnosis. Best Pract. Res. Clin. Obs. Gynaecol..

[28] Bordas C., Kovacs A., Pal B. (2015). The M-current contributes to high threshold membrane potential oscillations in a cell type-specific way in the pedunculopontine nucleus of mice. Front. Cell. Neurosci..

[29] Fellows R.P., Bangen K.J., Graves L.V., Delano-Wood L., Bondi M.W. (2022). Pathological functional impairment: Neuropsychological correlates of the shared variance between everyday functioning and brain volumetrics. Front. Aging Neurosci..

